# Mathematical model of salt cavern leaching for gas storage in high-insoluble salt formations

**DOI:** 10.1038/s41598-017-18546-w

**Published:** 2018-01-10

**Authors:** Jinlong Li, Xilin Shi, Chunhe Yang, Yinping Li, Tongtao Wang, Hongling Ma

**Affiliations:** 1 State Key Laboratory of Geomechanics and Geotechnical Engineering, Institute of Rock and Soil Mechanics, Chinese Academy of Sciences, Wuhan, 430071 Hubei China; 2 University of Chinese Academy of Sciences, Beijing, 100049 China

## Abstract

A mathematical model is established to predict the salt cavern development during leaching in high-insoluble salt formations. The salt-brine mass transfer rate is introduced, and the effects of the insoluble sediments on the development of the cavern are included. Considering the salt mass conservation in the cavern, the couple equations of the cavern shape, brine concentration and brine velocity are derived. According to the falling and accumulating rules of the insoluble particles, the governing equations of the insoluble sediments are deduced. A computer program using VC++ language is developed to obtain the numerical solution of these equations. To verify the proposed model, the leaching processes of two salt caverns of Jintan underground gas storage are simulated by the program, using the actual geological and technological parameters. The same simulation is performed by the current mainstream leaching software in China. The simulation results of the two programs are compared with the available field data. It shows that the proposed software is more accurate on the shape prediction of the cavern bottom and roof, which demonstrates the reliability and applicability of the model.

## Introduction

The usage of natural gas resources strongly fluctuates according to the season, while the gas well production is stable^1,2^. To balance the mismatch in gas supply and demand, the underground gas storage (UGS) has been used effectively as peak shaving means for nearly a century^3–5^. As one of the UGSs, salt cavern gas storage is constructed by cavern leaching in the sedimentary formation of salt rock, which formed from the drying up of enclosed salt lakes and seas^6^. Using one or more drilled wells, fresh water is injected into the salt formation to dissolve the salt deposits, brines are pumped out, and a cavern is developed gradually^7^. A salt cavern can be leached to hundreds of thousands of cubic meters, and it is an excellent choice for gas or oil storage, due to the good creep property, self-healing characteristics and extremely low permeability rate of salt rock^8–12^. There are 74 salt cavern UGSs in the world and the working gas volume is about 1.62 × 10^10^ m^3^, according to the research of Ozarslan^13^.

During the construction of salt cavern UGSs, the shapes of the caverns are the most important concern for the safety and stability of the cavern, which are always the primary considered issues^14,15^. According to the literatures^16–18^, an ellipsoidal shape is the most optimal structure for cavern stability. To ensure the salt cavern grows into the ideal shape, researchers have conducted many studies on the design and control of cavern shape during leaching. Duire and Jessen^19^ studied the solution mechanism of salt rock by laboratory experiments. They proposed an experience equation of the solution rate of salt rock. Nolen *et al*.^20^ designed a new digital program to simulate the cavern leaching process, which has been used successfully for years in Germany. The program took time, produced brine concentration and increased cavern volume as calculating parameters. Meanwhile, a certain amount of insolubles is considered. Reda and Russo^21^ conducted three salt cavity leaching experiments in the laboratory. They kept the cavity pressurized to actual cavern pressure and recorded the brine salinity and salt-wall recession. The experimental results are in good agreement with the numerical predictions. O’Hern *et al*.^22^ provided an experimental data record of measuring the leaching rates of salt walls. They found the dividing line between upper and lower regions of brines, roughly above and below the fresh water injection point. Li *et al*.^23^ set up a model to simulate the repair process of irregularly shaped salt cavern by re-leaching under gas. They conducted indoor experiments and field tests, the simulation results coincide well with the experimental results and field data, while the simulation of insolubles accumulation was not mentioned.

**Figure 1:**
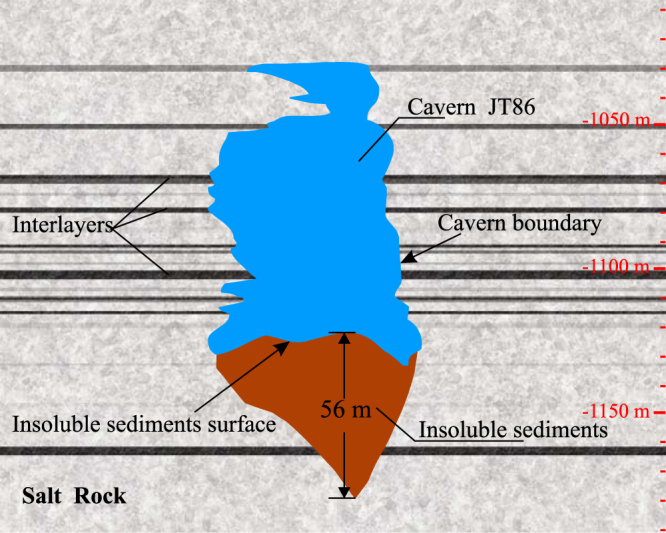
Insoluble sediments of Cavern JT86 of Jintan UGS in Jiangsu province, China.

However, most of these researches only considered the case of cavern form control in pure or low- insoluble salt rock. For their target salt caverns are mostly constructed in salt dome or thick salt formations, where the content of water-insoluble substances is very low and the development of the cavern is less affected. However, in many places, the salt formations contain significant amounts of water-insoluble substances, such as mudstone, sandstone, gypsum and limestone^24,25^. Especially in China, where most salt formations have interbedded water-insoluble interlayers, the salt beds are thin and the content of insoluble substances are higher than 20%^26^. Take an example, cavern JT86 (Fig. 1) is one of the completed salt caverns in Jintan UGS of Jiangsu province, China. There are many insoluble interlayers in the salt formations, and the average insolubles content of the salt formations around the cavern is about 21.6%. During leaching, the insoluble substances detached from the salt wall, and accumulated on the cavern bottom as loose insoluble particles with larger volume. The volume content of the insoluble sediments in this cavern reached about 35%, and the cavern bottom rose about 56 meters. Actually, the bottom of the cavern boundary is actually the surface of the insoluble sediments. For most Chinese salt formations, the content of insoluble substances is too high to ignore, the accumulation of the insolubles has greatly limit the lower cavern growth and decreased cavern capacity^27^.

Little research has been performed on the construction of salt cavern in these high-insoluble salt formations. Li *et al*.^27^ proposed a prediction model of the insolubles accumulation shape during leaching in salt formations with high content of insolubles. However, the model is two-dimensional and the insolubles accumulation is not coupled with the development of cavern shape. Zheng *et al*.^28^ discussed the water-soluble mechanism of thick interlayers in salt cavern. They proposed that the collapse of interlayers would connect the salt deposits above and below, and consequently enlarge cavern volume. A prediction model of the collapse of the interlayers was presented, whereas the solution mechanism of salt rock was not mentioned. The most commonly used cavern leaching software in China, “WinUbro”, has no completed insolubles calculation module^29^. All of the simulated caverns have a flat bottom, which does not match the reality that the cavern bottom shapes are variable^30^. The deviation of the cavern bottom results in the inconformity of total cavern shape, volume and development. Thus the salt cavern has to be detected by sonar and corrective measures need to be taken after each leaching stage, which brings great schedule and financial pressure.

For the further development of the construction of salt cavern gas storage in China, this paper proposes a prediction model of the single-well cavern leaching process in high-content insolubles salt formations. The salt-brine mass transfer rate is introduced to be related to the brine concentration, salt-brine contact angle and temperature. Based on the law of conservation of mass and reasonable assumptions, the calculation equations of cavern radius, brine concentration, flow rate, cavern volume and insolubles height are deduced. According to the particle packing theory, the re-distribution equation of the insoluble sediments is derived. Using the finite difference method, a VC++ computer program is developed and the numerical solution of the model can be obtained. Two salt caverns in Jintan UGS are selected and the actual leaching processes are simulated by the developed program and by the current software WinUbro. The comparison of the simulation results and the field data demonstrates the validity and accuracy of the proposed model.

## Methods

**Figure 2:**
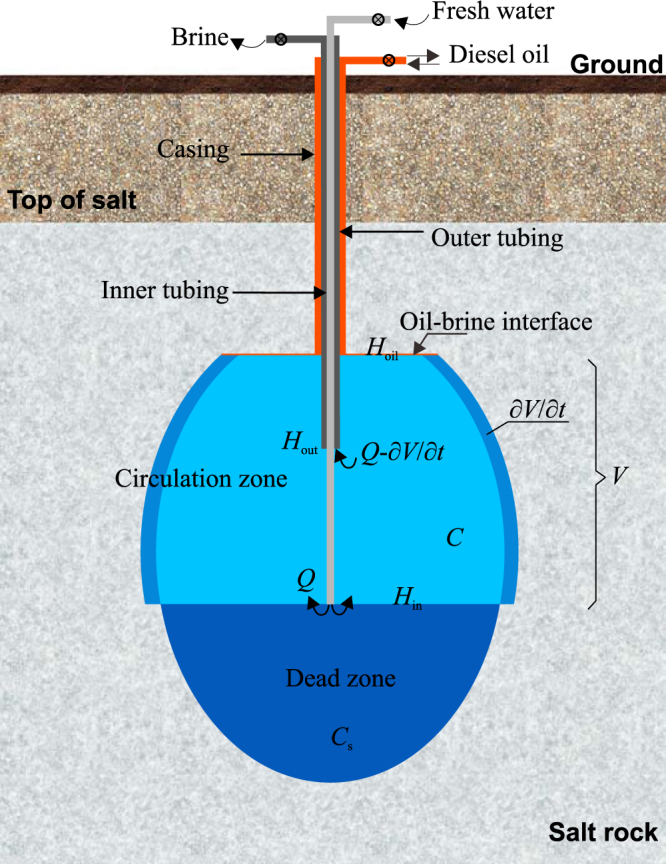
Sketch of the direct leaching mode.

In the cavern leaching process, two concentric tubings are placed into the leaching cavern through a casing pipe (Fig. 2). The casing pipe is the flow passage of blanket material, usually diesel oil, which is used to protect the upper salt from being dissolved. The inner and outer tubings are used for water injection and brine discharge. The depth of the inner and outer tubings, the injection flow rate, and the oil-brine interface depth are adjusted in each leaching stage to obtain an ideal cavern shape. The fresh water can be injected through inner or outer leaching tubing, when the leaching process can be distinguished into “direct leaching mode” and “diverse leaching mode”. In this section, the equations of the developing cavern boundary of the two modes are derived respectively, and the control equations of the insolubles sediments surface are deduced.

### Mathematical model of cavern boundary development

#### Direct leaching mode

Assume that the inner tubing orifice is placed at depth *H*_in_, outer tubing is at *H*_out_, and oil-brine interface is at *H*_oil_ (Fig. 2). Fresh water is injected through inner tubing in the direct leaching mode, the flow rate is *Q*. In most cases, the brine concentration of the circulation zone between *H*_in_ and *H*_oil_ can be assumed as uniform during the direct leaching period^22,23^. For the water injection tube is at the bottom of the circulation zone, the injected fresh water is far lighter than the brines and it will rise. While the high saturated brines generated by the dissolution of cavern top and cavern wall are heavy and they will sink. The up flow of fresh water and the down flow of brines will be fully blended, thus the concentration of the brines in the circulation zone can be assumed as *C* in this mode. Since there are no fresh water flowed into the dead zone under *H*_in_, the brines in the dead zone will keep saturated.

Taking the whole circulation zone as a control volume, the mass of salt is conserved,

1 $${\rho }\,\frac{\partial {V}}{\partial {t}}(1-{u})+(-\frac{\partial {{V}}_{{d}}}{\partial {t}}){{C}}_{{s}}-({Q}-\frac{\partial {V}}{\partial {t}}-\frac{\partial {{V}}_{{d}}}{\partial {t}}){C}=\frac{\partial ({VC})}{\partial {t}}$$

where, *ρ* is the desity of salt rock, *V* is the volume of the circulation zone, *u* is the content of insolubles in the salt formation, *V*_d_ is the volume of the dead zone under *H*_in_, *C*_*s*_ is the concentration of the saturated brines in the dead zone, *Q* is the flow of injected water, *C* is the concentration of the brines in the circulation zone, *t* is time.

The first term of Eq. (1) is the mass of the dissolved salt, the second term is the up flow of the dead zone due to the accumulation of insoluble sediments, and the third term is the mass of salt contained in the brine discharge through outer tubing.

$\frac{\partial {V}_{d}}{\partial t}$ is the volume change of the dead zone, causing by the accumulation of the insolubles contained in the salt formations. It can be calculated by,

2 $$\frac{\partial {V}_{{\rm{d}}}}{\partial {t}}=-\frac{\partial {\rm{V}}}{\partial {t}}{uf}$$

where, *f* is the expansion coefficient of insoluble substances in brine.

The volume of the circulation zone *V* can be calculated by,

3 $${V}={\int }_{0}^{2{\boldsymbol{\pi }}}{\int }_{{H}_{{\rm{in}}}}^{{H}_{{\rm{oil}}}}\frac{1}{2}{R}^{2}{\rm{d}}h\,{\rm{d}}\theta $$

where, *H*_oil_ is the depth of oil-gas interface, *H*_in_ is the depth of inner leaching tubing, *R* is the cavern radius, *h* is height, *θ* is circumferential angle.

The cavern radius *R* is related to the dissolution rate of cavern wall,

4 $$\frac{\partial {R}}{\partial {t}}=\frac{{\omega }}{\sin \,\alpha }$$

where, *ω* is the dissolution rate of the salt cavern wall, *α* is the angle between the normal direction of cavern wall and vertical upward direction, or salt-brine contact angle for short.

*ω* is actually the mass transfer rate between salt and brine, which is mainly related to the brine concentration, salt-brine contact angle, and temperature of brines^31^. According to the research of Xiao *et al*.^32^, *ω* can be calculated by an empirical formula. In addition, if the salt wall is covered by insoluble sediments, the salt is not exposed to the brines, and then the dissolution will stop.

5 $$\omega =\{\begin{array}{cc}{\omega }_{0}\times ({C}_{s}-C)\mathrm{ln}\,\frac{{T}^{0.44}}{e}{(\sin \alpha )}^{0.25} & (\alpha  > 0)\\ {\omega }_{0}\times ({C}_{s}-C)\mathrm{ln}\,\frac{{T}^{0.44}}{e}\frac{90-\alpha }{90} & (\alpha  < 0)\\ 0 & (\alpha \,{\rm{is}}\,{\rm{not}}\,\text{exist})\end{array}$$

where, *T* is the temperature of the brine, *e* is the natural logarithm, *ω*_0_ is the dissolution rate of pure salt in fresh water, which can be easily measured in laboratory.

*α* can be calculated by,

6 $${\alpha }={\rm{arccot}}\frac{\partial {R}}{\partial {h}}$$

#### Diverse leaching mode

**Figure 3:**
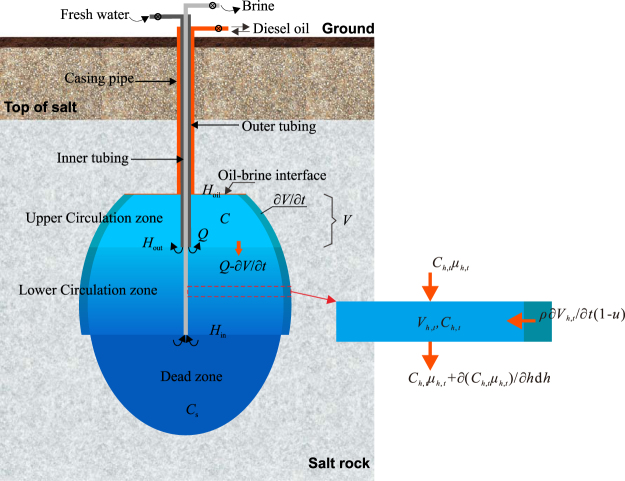
Sketch of the diverse leaching mode.

During the diverse leaching period, the fresh water is injected through the higher outer tubing, while the brine is discharged through the lower inner tubing (Fig. 3). The circulation zone is divided into two parts, upper circulation zone above *H*_out_ and lower circulation zone under *H*_out_. The distribution of the brine concentration in the upper circulation zone is just like that in the circulation zone of the direct leaching mode. Taking the upper circulation zone as a control volume, and the mass conservation equation of this part can be written as,

7 $${\rho }\frac{\partial {V}}{\partial {t}}(1-{u})-({Q}-\frac{\partial {V}}{\partial {t}})C=\frac{\partial ({VC})}{\partial {t}}$$

where, *V* can be calculated by,

8 $${V}={\int }_{0}^{2{\pi }}{\int }_{{{H}}_{{\rm{out}}}}^{{{H}}_{{\rm{oil}}}}\frac{1}{2}{{R}}^{2}{\rm{d}}{h}\,{\rm{d}}{\theta }$$

where, *H*_out_ is the depth of outer leaching tubing orifice.

The first term of Eq. (7) is the mass of the dissolved salt in the upper circulation zone, and the second term is the mass of salt contained in the brines discharged into the lower circulation zone. The term on the right side of Eq. (7) is the salt mass change of the control volume. Compared with Eq. (1), the up flow of the saturated brines in the dead zone due to the accumulation of insoluble sediments is discharged from the lower inner tubing, thus the related term does not appear in Eq. (7).

Due to the injection of fresh water, the volume of the brines in the upper circulation zone expands and the extra brines flow down to the lower circulation zone. The brines in the lower zone are pushed downward, dissolving salts along the way, and finally discharged through the inner tubing. The lower circulation zone can be divided into numerous horizontal micro sections, the heights of which are d*h*. Due to the horizontal stratification of brines, it can be assumed that the brines concentrations in each micro section are uniform^23^. The mass conservation equation of the micro sections can be expressed as,

9 $$\frac{\partial ({{C}}_{{h},{t}}{{\mu }}_{{h},{t}})}{\partial {h}}{\rm{d}}{h}+{\rho }\frac{\partial {{V}}_{{h},{t}}}{\partial {t}}(1-{u})=\,\frac{\partial ({{C}}_{{h},{t}}{{V}}_{{h},{t}})}{\partial {t}}$$

where, *h* is depth as well as the code name of the micro section whose depth is *h*, *C*_*h*,t_ is the brine concentration of section *h*, *μ*_*h*,*t*_ is the vertical flow rate of section *h*, *V*_*h*,*t*_ is the volume of section *h*.

*V*_*h*,*t*_ can be written as,

10 $${{V}}_{{h},{t}}={\int }_{0}^{2{\pi }}{R}{\rm{d}}{\theta }{\rm{d}}{h}$$

Since the brine cannot be compressed, the brine flow *μ*_*h*,*t*_ in Eq. (9) can be calculated by the volume conservation equation

11 $$\{\begin{array}{cc}{\mu }_{h,t}=Q-\frac{\partial V}{\partial t} & (h=\,{H}_{{\rm{out}}})\\ \frac{\partial {\mu }_{h,t}}{\partial h}=\frac{\partial {V}_{h,t}}{\partial t} & ({H}_{{\rm{in}}} < h < {H}_{{\rm{out}}})\end{array}$$

### Mathematical model of insoluble sediments surface

During cavern leaching, with salt dissolved into brines, the insoluble substances contained in the salt lose support. They detach from the cavern wall and accumulate on the cavern bottom. The salt cavern development is closely related to the insoluble sediments in the cavern. For the accumulation of the insolubles will cover the lower cavern wall and protect them from being dissolved. Furthermore, the bottom of a complete cavern is actually the surface of the insoluble sediments. In this section, the accumulation of the insoluble substances will be discussed.

Falling from the cavern wall, an insoluble particle will finally settle within a distance to the projection of its starting point on the cavern bottom. According to the research of Li *et al*.^27^, this distance is basically proportional to the height of the falling path, as shown in Eq. (12).

12 $${R}_{{\rm{fall}}}=D{H}_{{\rm{fall}}}$$

where, *R*_fall_ is the maximum distance from the position of a fallen insoluble particle to the projection of its starting point on the cavern bottom, *H*_fall_ is the height of the falling path of the insoluble particle, *D* is the scattering coefficient between *R*_fall_ and *H*_fall_, the value of *D* can be obtained by laboratory experiments.

**Figure 4:**
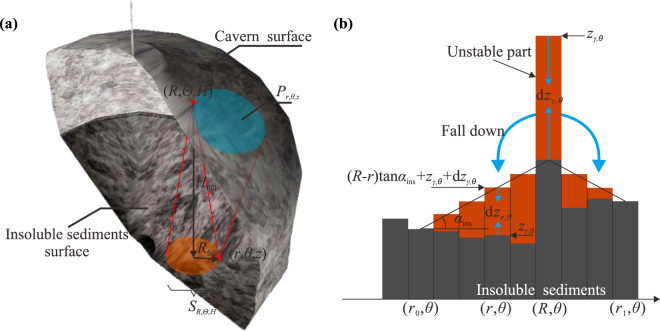
Accumulation of insoluble substances on the cavern bottom. (**a**) Fallen. (**b**) Redistribution.

In other words, the insoluble substances, fallen from point (*R*, *Θ*, *H*), will accumulate within a radius around the projection of (*R*, *Θ*, *H*) on the cavern bottom (insoluble surface), as shown in Fig. 4(a). We can assume a set *P*_*r*,*θ*,*z*_ to represent a number of cavern wall points, the insolubles from where may fall onto point (*r*, *θ*, *z*) on the insoluble sediments surface. Every point in the set will satisfy the following equation,

13 $$\sqrt{{R}^{2}+{r}^{2}-2Rr\,\cos (\Theta -\theta )} < D(H-z)\,(R,\Theta ,H)\in {{\rm P}}_{r,\theta ,z}$$

where, *P*_*r*,*θ*,*z*_ is the set of the cavern wall points, the insolubles from where may fall onto (*r*, *θ*, *z*).

Assuming an even distribution of the insolubles in the settle circle, the height change of insoluble sediments surface can be calculated by,

14 $$\frac{\partial z}{\partial t}=\sum _{(R,\Theta ,H)\in {{\rm P}}_{r,\theta ,z}}\frac{{\epsilon }{U}_{R,\Theta ,H}\partial {V}_{R,\Theta ,H}}{\pi {[D(H-z)]}^{2}\partial t}$$

where, *U*_*R*,Θ,*H*_ is the insoluble content of the micro-element on point (*R*, Θ, *H*), $\tfrac{\partial {V}_{R,\Theta ,H}}{\partial t}$ is the volume variation of the micro-element on point (*R*, Θ, *H*), *∈* is the expansion coefficient of the insoluble substances in brine, which has considered the effects of broken expansion and compaction.

According to the particle packing theory, the tangential angle of the insolubles surface should be always less than the repose angle of insoluble particles. To simplify the calculation, we only consider the tangential angle limit on the radial direction and circumferential direction, which can be expressed as,

15 $$\{\begin{array}{c}{\rm{abs}}(\frac{\partial z}{\partial r}) < \,\tan \,{\alpha }_{{\rm{ins}}}\,\\ {\rm{abs}}(\frac{\partial z}{\partial \theta }) < \,\tan \,{\alpha }_{{\rm{ins}}}\,\end{array}$$

where, *α*_ins_ is the angle of repose of the insoluble particles in brines.

For all of the points on the insolubles surface which do not meet Eq. (15), the height of the insoluble sediments should be adjusted. For example, in the radial direction, the height of the insoluble sediments on point (*γ*, *θ*) is *z*_*γ*,*θ*_, it is too high and the unstable part fall down, as shown in Fig. 4(b). The height of the insolubles on this point decreases, while the insoluble heights of neighboring lower points increase. The volume conservation equation of the insoluble sediments can be expressed as,

16 $$\frac{{\rm{d}}({\gamma }^{2}){\rm{d}}\theta }{2}{\rm{d}}{z}_{\gamma ,\theta }=\sum _{r={r}_{0}}^{{r}_{0}\le r\le {r}_{1},\,r\ne \gamma }\frac{{\rm{d}}({r}^{2}){\rm{d}}\theta }{2}{\rm{d}}{z}_{r,\theta }$$

where, *z*_*γ*,*θ*_ is the insolubles height on point (*γ*, *θ*), z_*r*,*θ*_ is the insolubles height on point (*r*, *θ*), *r*_0_ and *r*_1_ respectively are the radii of the leftmost and rightmost points affected by the fallen insolubles from (*γ*, *θ*).

The tangential angle of the re-distributed insolubles will be equal to *α*_ins_, thus

17 $${z}_{r,\theta }+{\rm{d}}{z}_{r,\theta }=r\,\tan \,{\alpha }_{{\rm{ins}}}+{z}_{\gamma ,\theta }+{\rm{d}}{z}_{\gamma ,\theta }\,({r}_{0}\le r\le {r}_{1})$$

On point (*r*_0_, *θ*), the height of insolubles does not change after redistribution,

18 $${\rm{d}}{z}_{{r}_{0},\theta }=0$$

The redistribution of the insoluble sediments is similar in the circumferential direction, and we can easily obtain the adjustment height in the circumferential directions. Thus, considering the redistribution, Eq. (14) should be re-written as,

19 $$\frac{\partial z}{\partial t}=(\sum _{(R,\Theta ,H)\in {{\rm P}}_{r,\theta ,z}}\frac{{U}_{R,\Theta ,H}\partial {V}_{R,\Theta ,H}}{\pi {[D(H-z)]}^{2}\partial t})+{\rm{d}}{z}_{r,\theta }+{\rm{d}}{z}_{r,\theta }^{\prime} $$

where, d*z*_*r*,*θ*_ and ${\rm{d}}{z}_{r,\theta }^{\prime}$ respectively are the height changes of the insolubles in the radial and circumferential directions at point (*r*, *θ*).

### Software implementation for numerical solution

**Figure 5:**
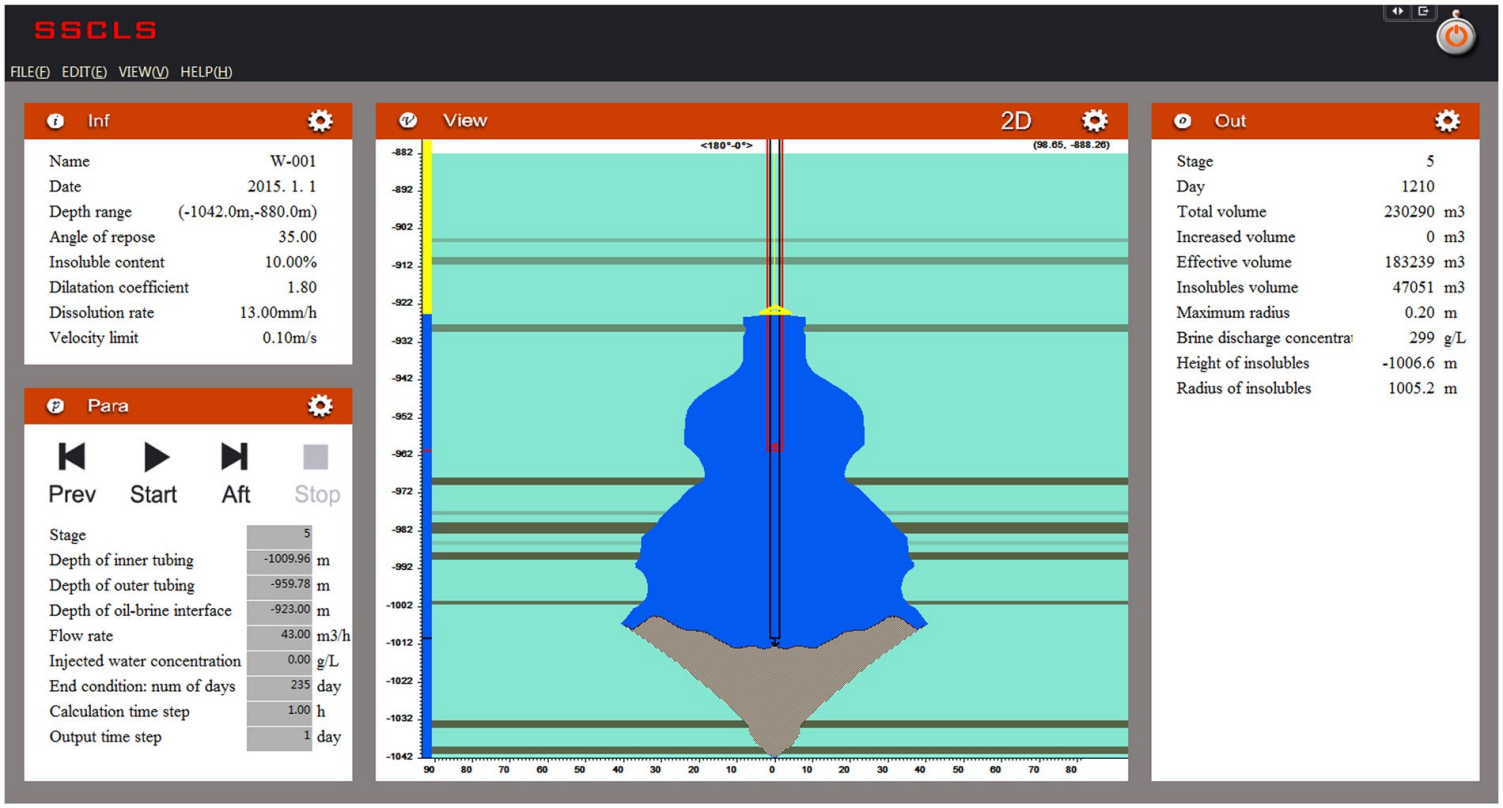
Screenshot of SSCLS (Single-well Salt Cavern Leaching Simulation V1.0).

Eqs (1–19) provides the theoretical formulae for the two modes of the cavern leaching process. Given the technological parameters and the initial values of the control variables, the developing cavern boundary, brine concentration and insoluble sediments surface can be calculated. Based on the equations, a computer program “Single-well Salt Cavern Leaching Simulation V1.0”, “SSCLS” for short, is developed to solve the numerical solution of the model. Using finite difference method, SSCLS is written in the VC++ language, with independent operation interface as shown in Fig. 5. There are five function modules in SSCLS: formation information and sonar data import, technical parameters input, flow/concentration field calculation, cavern boundary calculation, and insoluble sediments surface calculation.

**Figure 6:**
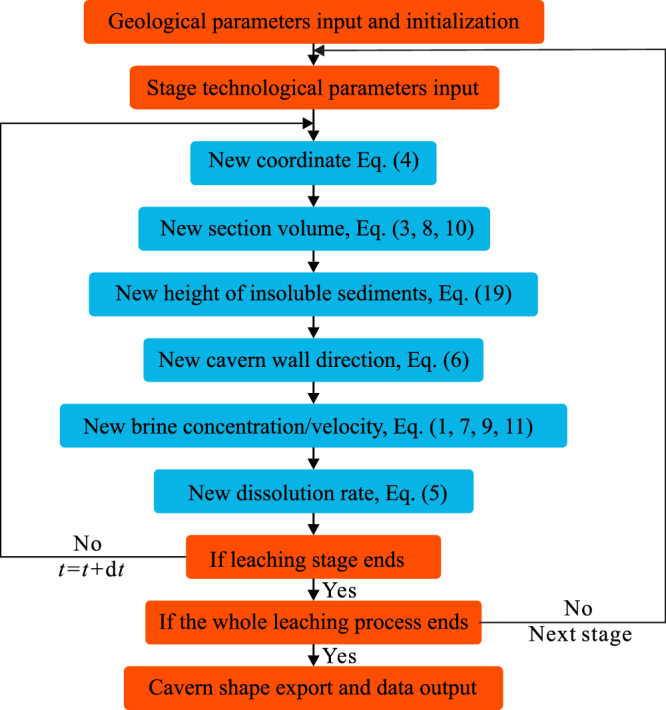
Flow chart of the simulation of cavern leaching by SSCLS.

The cavern is divided into numerous micro-sectors, with different serial number, cylindrical coordinate, volume, brine flow velocity, brine concentration, salt-brine contact angle, and insolubles content. All of these sector information are stored and calculated in array in every time step, until the end of the leaching process. In the cavern system, a grid of the insoluble sediments surface is set up. The height of the insolubles on each grid is calculated and amended after the change of cavern shape. The calculation flow chart of the whole simulation process is shown in Fig. 6.

### Data availability

The data generated during and/or analysed during the current study are available from the corresponding author on reasonable request.

## Results

Since the whole construction period is quite long (3–7 years), we cannot verify the proposed model by simulation-construction-comparison. Second best, we can simulate the leaching process of a complete salt cavern, using the practical geological and technological parameters, and then compare the simulation results with the actual cavern shape to verify the model. Two complete salt caverns of Jintan UGS in Jiangsu province, a regular shaped cavern JT52 and an irregular shaped cavern JT103, are selected for simulation. The same simulation will be performed by WinUbro, the most commonly used cavern leaching software in China^33^. The simulation results are shown in this section.

### Simulation results of the regular salt cavern JT52

Cavern JT52 is one of the earliest constructed salt caverns of Jintan UGS in Jiangsu, China. The buried depth range of the salt formations is between −1140 m and −990 m, the average salt purity is about 78.8%. There are about 7 insoluble interlayers in the formation, the major components of the interlayers are mudstone and gypsum. Cavern JT52 was built between 2003 and 2010, the volume of the complete cavern is about 186,675 m^3^ and the depth range is between −1089.5 m and −1015.4 m. The whole cavern construction process has 7 direct leaching stages with different technological parameters, including inner/outer tubing depth, oil-brine interface depth, stage time and water injection rate, as shown in Table 1.

**Table 1:** Technological parameters of cavern JT52.

Stage	Leaching mode	Inner tubing depth (m)	Outer tubing depth (m)	Oil pad depth (m)	Stage time(d)	Injection rate (m^3^/h)
1	Direct	−1129.2	−1111.9	−1095.2	38	46.2
2	Direct	−1117.1	−1065.5	−1053.3	92	48.6
3	Direct	−1113.4	−1065.0	−1051.1	420	69.1
4	Direct	−1099.1	−1035.7	−1035.2	199	66.3
5	Direct	−1085.2	−1025.5	−1025.4	241	62.8
6	Direct	−1080.1	−1016.2	−1015.4	190	65.2
7	Direct	−1080.1	−1016.2	−1015.4	112	64.4

**Figure 7:**
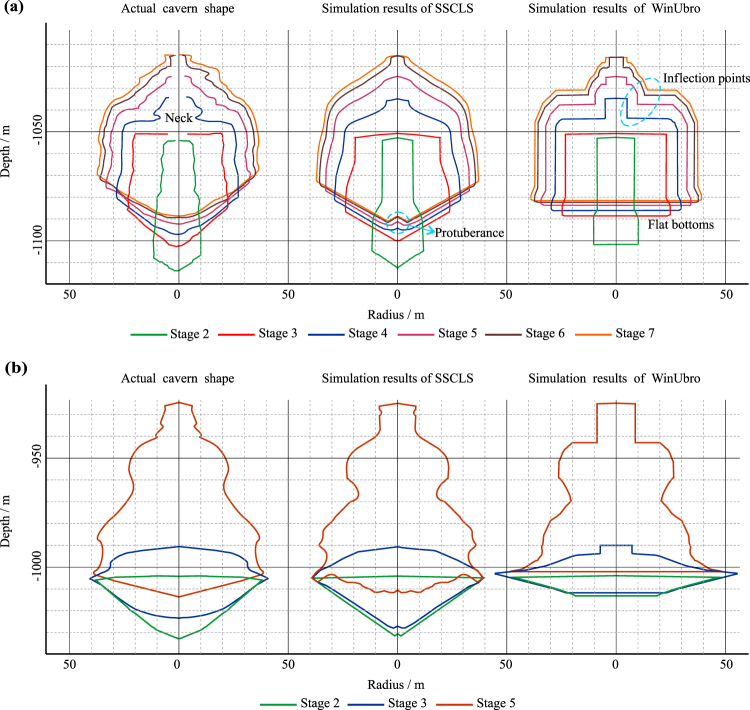
Comparison of the cavern shapes of the simulation results and sonar imaging data. (**a**) Cavern JT52. (**b**) Cavern JT103.

The practical leaching parameters and geological data are imported into SSCLS and WinUbro, where the seven leaching stages are simulated. The comparison of the simulation results and the actual cavern shape is shown in Fig. 7(a). Since cavern JT52 is roughly axisymmetric, only the vertical section in the north-south direction is compared. Since there is no sonar data of the first leaching stage, only cavern shape comparison of stages 2–7 are shown.

### Simulation results of the irregular salt cavern JT103

Cavern JT103 is a lately completed salt cavern in Jintan UGS, started in 2012 and completed in 2016. The buried depth range of the salt formations is from −1050 m to −880 m, with 12 insoluble mudstone interlayers. The average salt purity is about 84.5%. The volume of the complete cavern is about 181275 m^3^ and the depth range is between −1013.4 m and −923.4 m. The whole cavern construction process has 1 direct leaching stages and 4 diverse leaching stages, with different technological parameters (Table 2).

**Table 2:** Technological parameters of cavern JT103.

Stage	Leaching mode	Inner tubing depth (m)	Outer tubing depth (m)	Oil depth (m)	Stage time(d)	Flow rate (m^3^/h)
1	Direct	−1043.0	−1015.0	−1005.4	226	50.2
2	Diverse	−1038.2	−1015.0	−1005.3	229	47.2
3	Diverse	−1036.7	−1003.9	−991.2	218	52.4
4	Diverse	−1022.8	−992.9	−960.0	302	79.1
5	Diverse	−1009.9	−959.8	−923.1	235	43.8

The leaching processes are simulated by SSCLS and WinUbro at the same time, using the actual geological and technological parameters. The simulated cavern shapes of the two programs and the actual cavern shapes (average radii) are drawn in Fig. 7(b). Since there is no sonar data of the 1^st^ and 4^th^ leaching stage, only cavern shape comparison of stages 2, 3, 5 are shown.

## Discussion

### Comparison of the simulation results and the field data of JT52

**Figure 8:**
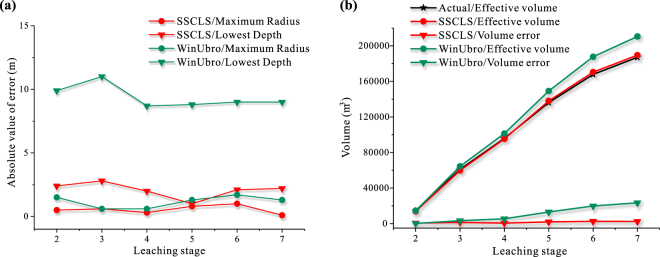
Comparison of the main parameters of the simulation results and sonar imaging data of cavern JT52. (**a**) Absolute value of errors of maximum radius and lowest depth. (**b**) Effective volume and error.

As shown in Fig. 7(a), the lateral dimensions of the simulated caverns by using the two simulators (SSCLS and WinUbro) are both fairly accurate. The maximum radius errors of the two simulations are less than 2 meters (Fig. 8(a)), accounting for about 5% of the maximum radius, which can meet the requirements of actual engineering. However, the simulations are quite different on the cavern top and bottom. The cavern bottom shapes of the simulation of SSCLS are closer to the actual shapes, the errors of the lowest depth are about 1–3 meters (Fig. 8(a)), which is a pretty high accuracy. Since WinUbro has no function module about the insolubles accumulation, the cavern bottoms are handled as flat, which have a large deviation from the actual shapes. There are 8–11 meters errors between the lowest depth of WinUbro’s simulations and the actual caverns. As for the top part of the cavern, the SSCLS simulated shapes basically coincide with the actual shapes, while the WinUbro’s simulated cavern has an inflection point after stage 4 (Fig. 7(a)). The reason is that, the control points of the cavern boundary of SSCLS move in the normal direction, which is the same as that of the actual cavern wall. The cavern control points of WinUbro moves in the radial direction, resulting in the rough of the cavern top boundary. Due to the perfectly match on the cavern top and bottom, the volume error of the simulation of SSCLS (about 2400 m^3^) is much less than that of WinUbro (about 23,000 m^3^), as shown in Fig. 8(b).

After stage 4, a protuberance appeared in the middle of the cavern bottom of the SSCLS simulated cavern (Fig. 7(a)), for the insolubles in the top center of the cavern fell and accumulate on the bottom center. However, the actual cavern bottom is pretty smooth in the middle part. The reason is that, fresh water was injected through the lower inner tubing near the insoluble surface, the insoluble sediments were re-distributed due to the injection flow. For example, the inner tubing was placed at −1099.1 m at the 4^th^ stage, when the middle point of the insoluble surface was at −1098.8 m. The injection flow smoothen the cavern bottom, which is not considered in the proposed model and SSCLS at this stage.

In addition, the actual cavern shape has a “neck” at depth −1044 m in the 4^th^ stage, which probably results from the effect of the insoluble interlayers. The SSCLS simulated cavern shape is pretty smooth at −1044 m, for the proposed model only considered the simulation of cavern shape change due to salt dissolution. The dissolution, soften and collapse of the insoluble interlayers is not included. The effect of these insoluble interlayers on the cavern development might be discussed in the next step of our work.

### Comparison of the simulation results and the field data of JT103

**Figure 9:**
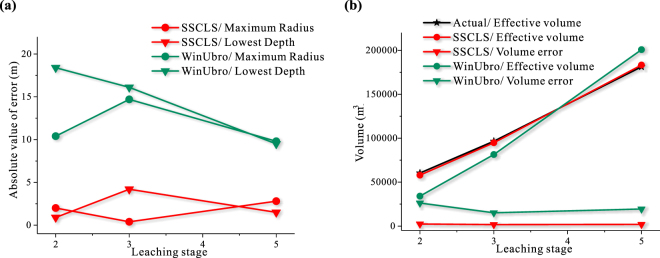
Comparison of the main parameters of the simulation results and sonar imaging data of cavern JT103. (**a**) Absolute value of cavern radius and lowest depth. (**b**) Effective volume and error.

As shown in Fig. 7(b), the SSCLS simulated cavern shapes basically coincide with the actual shapes of each stage. The radius errors are less than 2 meters (Fig. 9(a)), about 5% of the maximum cavern radius. The errors of the lowest cavern depth are less than 1.6 m in stage 2 and 5, about 1.8% of the cavern height. However, in stage 3, the lowest cavern depth error is about 9 meters (Fig. 9(a)). The simulated cavern bottom is lower and steeper than the actual cavern bottom. The inner tubing was placed at −1036.3 m at the 3^rd^ stage, buried under the middle point of the insoluble surface, which rise from −1031.8 m to −1022.6 m. The brine discharge current around the inner tubing washed the insolubles from both sides to the center part. As mentioned in the comparison of cavern JT52, the impact of the flow field on the insoluble distribution was not considered in SSCLS, resulting in the difference between the simulation and actual cavern bottom in stage 3. The effect of the injection flow on the redistribution of insoluble sediments might be discussed in our next step work. However, the effect does not change the total volume of the cavern. The effective volume of the simulation results of SSCLS still has a high accuracy, the final volume error is less than 3000 m^3^ (Fig. 9(b)), about 1.6% of the total cavern volume.

The WinUbro simulated shapes have large deviation in the lower part of the cavern. In the second stage, the actual cavern is roughly obconical, the flat bottom processing mode of WinUbro results in large errors in maximum radius, volume and lowest point of the cavern. The error accumulates in the following stages. In the final stage, the radius error is 10 m, the volume error is about 19,000 m^3^, and the lowest depth error is 9.5 m (Fig. 9).

In general, the proposed model can accurately predict the cavern development and insolubles accumulation during leaching for gas storage within engineering precision limitation. Compared with WinUbro, the simulation results of SSCLS are more consistent with the actual shape on cavern bottom and top.

## Conclusions

(I) A mathematical model is proposed to predict the cavern development during cavern leaching for gas storage. The salt-brine mass transfer rate is introduced to be related to the brine concentration, salt-brine contact angle and temperature. The salt mass conservation is considered, and the couple equations of the cavern shape, brine concentration and flow rate of the direct and diverse leaching modes are deduced.
(II) According to the falling and accumulating rules of the insoluble substances in brines, the calculation equations of the surface of the insoluble sediments are introduced for the first time. The redistribution of the insoluble sediments is considered according to the particle packing theory. The amendatory control equations of the insoluble sediments surface are derived.
(III) A VC++ software SSCLS is developed to obtain the numerical solution of the proposed model. Using the actual geological data and technological parameters, the leaching processes of two salt caverns of Jintan UGS are simulated by SSCLS and by WinUbro, the most commonly used cavern leaching software in China. The simulation results of SSCLS coincide well with the actual cavern shape, and are more accurate on the shape prediction of the cavern bottom and top. It shows that the proposed model is reliable for the simulation of cavern leaching in China.
